# Comparison of Human Dermal Fibroblasts and HaCat Cells Cultured in Medium with or without Serum via a Generic Tissue Engineering Research Platform

**DOI:** 10.3390/ijms19020388

**Published:** 2018-01-28

**Authors:** Christopher Michael Gabbott, Tao Sun

**Affiliations:** Centre for Biological Engineering, Department of Chemical Engineering, Loughborough University, Epinal Way, Loughborough LE11 3TU, UK; c.gabbott@lboro.ac.uk

**Keywords:** 2D cell culture, 3D tissue culture, scale-down model, serum, fibroblast, HaCat cell

## Abstract

A generic research platform with 2-dimensional (2D) cell culture technology, a 3-dimensional (3D) in vitro tissue model, and a scaled-down cell culture and imaging system in between, was utilized to address the problematic issues associated with the use of serum in skin tissue engineering. Human dermal fibroblasts (HDFs) and immortalized keratinocytes (HaCat cells) mono- or co-cultured in serum or serum-free medium were compared and analyzed via the platform. It was demonstrated that serum depletion had significant influence on the attachment of HaCat cells onto tissue culture plastic (TCP), porous substrates and cellulosic scaffolds, which was further enhanced by the pre-seeded HDFs. The complex structures formed by the HDFs colonized within the porous substrates and scaffolds not only prevented the seeded HaCat cells from filtering through the open pores, but also acted as cellular substrates for HaCat cells to attach onto. When mono-cultured on TCP, both HDFs and HaCat cells were less proliferative in medium without serum than with serum. However, both cell types were successfully co-cultured in 2D using serum-free medium if the initial cell seeding density was higher than 80,000 cells/cm^2^ (with 1:1 ratio). Based on the results from 2D cultures, co-culture of both cell types on modular substrates with small open pores (125 μm) and cellulosic scaffolds with open pores of varying sizes (50–300 µm) were then conducted successfully in serum-free medium. This study demonstrated that the generic research platform had great potential for in-depth understanding of HDFs and HaCat cells cultivated in serum-free medium, which could inform the processes for manufacturing skin cells or tissues for clinical applications.

## 1. Introduction

Tissue engineering (TE) aims to produce bio-substitutes for the treatment of tissue injuries and organ failures [1,2,3]; and it is essential to further develop the proof-of-concept (PoC) studies into fully functional tissues for clinical applications [4,5]. However, this translational task from bench-top to bedside is hindered by the lack of sufficient mechanistic understanding of tissue formation due to the limitations associated with the current cell and tissue culture technologies [6,7,8]. For example, cutaneous wound healing has long been a magnet for tissue engineering, and new advanced TE products and technologies are usually introduced into this field. Nevertheless, there is still a lack of good limb salvage options for wound healing [2,9]. The aim of this study was to develop a generic research platform based on multiple cell and tissue culture technologies, which was then utilized to address the translational challenge associated with the use of serum in skin tissue engineering.

The expansion of human keratinocytes for clinical applications is often performed using feeder fibroblast cells and culture medium supplemented with animal derived products such as fetal bovine serum (FBS), which has a rich content of growth factors [10,11]. Apart from immune rejection, the use of FBS is associated with several problematic issues. For example, due to the high level of batch-to-batch variation and the presence of unidentified growth factors and other proteins, the media supplemented with serum are usually poorly defined [12]. Additionally, as 20–50% of the commercially available FBS products are virally contaminated, they can cause the transmission of virus and other pathogenic agents [13]. With the growing concerns of these problems, European regulatory authorities prefer that cells and tissues cultured for clinical purposes avoid the use of bovine and other animal derived products [14]; and research efforts have been focused on the culture of skin cells in serum-free medium [15,16,17]. Previously, we successfully cultured human dermal fibroblasts and normal human keratinocytes in serum-free medium using both two-dimensional (2D) cell culture [16] and a three-dimensional (3D) tissue culture system [17]. However, these commonly used cell and tissue culture technologies are associated with various limitations [6,7,8]. The traditional 2D cell culture is simple and cost-effective, but it is usually considered as a poor proxy [18,19]. Complex 3D tissue models have been developed to mimic the complicated in vivo microenvironments [20], but it has remained difficult to distinguish the regulatory functions of each individual architectural feature at different scales, as well as other biochemical and biomechanical properties during 3D tissue cultures [21,22]. Moreover, the data from the distinct 2D cell and 3D tissue systems are usually not comparable [18,19,20]. Therefore, to further advance our PoC level studies to produce functional skin cells or tissues for clinical applications, a new research strategy is required to obtain further mechanistic insights of the cultivated skin cells and tissues. To bridge the gap between the current 2D cell and 3D tissue culture technologies, we have recently developed a 3D cell culture and imaging system (3D CCIS) [22]. In this study, the 3D CCIS was combined with 2D cell culture technology and a 3D tissue culture model, to develop a generic research platform. The critical process parameters during the expansion of human dermal fibroblasts (HDFs) and immortalized human keratinocytes (HaCat cells) in medium with or without supplemented serum were then carefully examined and compared using this research platform, which not only provided in-depth understanding of the cultivated cells, but also informed the manufacturing of skin cells and tissues in serum-free medium as detailed in the following sections.

## 2. Results

### 2.1. 2D Cell Culture in Medium with or without Serum

**Figure 1:**
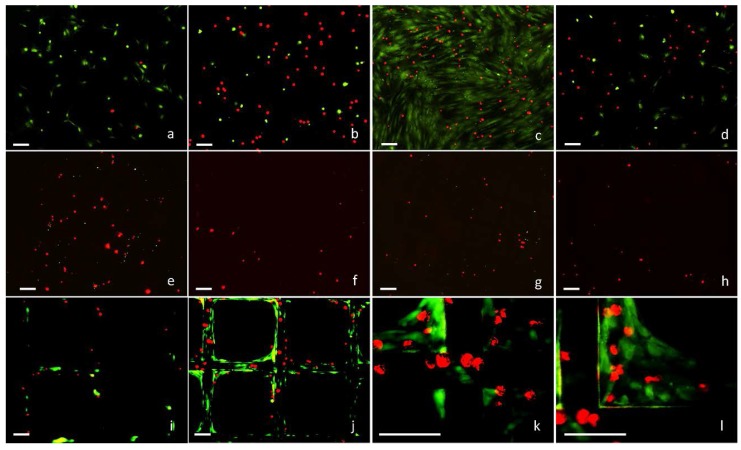
Fluorescent micrographs of HaCat cells and HDFs attached onto tissue culture plastic (TCP) or modular substrates in medium with or without serum. HDFs were seeded onto TCP (5 × 10^3^ cells/cm^2^), incubated for 40 min in medium (**a**) with or (**b**) without serum, or cultured for 5 days in medium (**c**) with or (**d**) without serum. HaCat cells in the same medium with or without serum were then seeded onto the same TCP surfaces (5 × 10^3^ cells/cm^2^) and incubated for 40 min. Aliquot of 200 μL HDF suspension (1 × 10^5^ cells/mL) was seeded onto each of the modular substrates with pore sizes of (**e**) 280 μm, (**g**) 420 μm in serum-free medium and cultured for 40 min, or seeded onto each of the modular substrates with the pore size of 420 μm and cultivated in medium with serum for (**i**) 0 day or (**j**–**l**) 5 days. The same volume (200 μL) of HaCat cell suspension (1 × 10^5^ cells/mL) was then seeded onto each of these substrates with the same medium, or onto new modular substrates with pore sizes of (**f**) 280 μm, (**h**) 420 μm in medium without serum as the control and incubated for 40 min. For fluorescent microscopy, HDFs and HaCat cells were stained with GREEN and RED cell trackers respectively. Scale bar = 100 μm.

**Figure 2:**
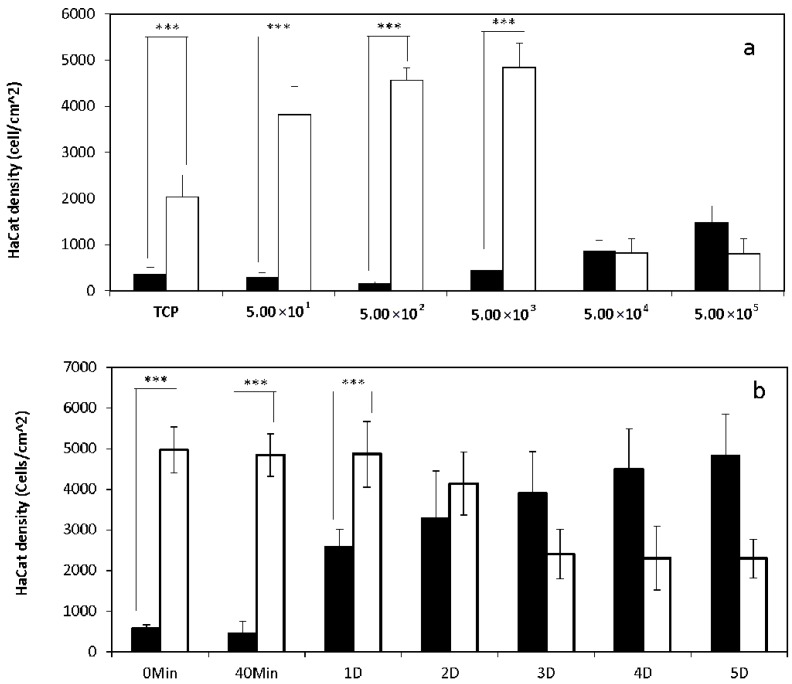
The influence of serum and HDFs on the attachment of HaCat cells onto tissue culture plastic (TCP). (**a**) HDFs stained with GREEN cell tracker were seeded onto TCP at different densities (5 × 10^1^, 5 × 10^2^, 5 × 10^3^, 5 × 10^4^, 5 × 10^5^ cells/cm^2^) in medium with (Black) or without (White) serum and incubated for 40 min. (**b**) HDFs stained with GREEN cell tracker were seeded onto TCP with the defined density of 5 × 10^3^ cells/cm^2^ in medium with (Black) or without (White) serum, and incubated for 0 and 40 min (Min), or cultured for 1 to 5 days (D). HaCat cells stained with RED cell tracker in medium with or without serum were added to the TCP with or without HDFs with the density of 5 × 10^3^ cells/cm^2^, and incubated for 40 min. Fluorescent micrographs were captured, and analyzed via image J to register the attached HaCat cells. (Results shown are mean value ± SD, *n* = 3). (*** *p* < 0.001).

HDFs stained with GREEN cell tracker were seeded onto tissue culture plastic (TCP) with different densities (50, 500, 5000, 50,000, 500,000 cells/cm^2^) in medium with or without supplemented serum, and incubated at 37 °C for 40 min. HaCat cells stained with RED cell tracker were then added onto these TCP (5000 cells/cm^2^) with the same medium, or seeded on their own onto separate TCP (5000 cells/cm^2^) as the control. After further incubated for 40 min, the samples were fixed, and fluorescent micrographs of the attached cells were captured (Figure 1a,b) and analyzed using Image J. As shown in Figure 2a, the attachment of HaCat cells onto TCP in the absence of serum was significantly higher than in the medium with serum, which was further enhanced by the pre-seeded HDFs with densities in the range of 50 to 5000 cells/cm^2^. However, when the HDF density was further increased to 50,000 and 500,000 cells/cm^2^, the number of attached HaCat cells decreased dramatically.

HDFs stained with GREEN cell tracker were seeded (5000 cells/cm^2^) onto TCP in medium with or without serum, incubated for 0 or 40 min, or further cultured for 1 to 5 days. HaCat cells stained with RED cell tracker were then seeded onto the same TCP surfaces (5000 cells/cm^2^) in the same medium. After a further incubation period of 40 min, the attached HaCat cells were registered via fluorescent microscopy (Figure 1c,d). As illustrated in Figure 2b, both the freshly seeded and the briefly cultured (1 day) HDFs in serum-free medium facilitated significantly more HaCat cell attachment than in medium with serum. Interestingly, as the culture time was further increased to 5 days, the impact of HDFs on HaCat cell attachment in serum-free medium dramatically declined to the bottom level. In comparison, the influence of HDFs on HaCat cell attachment in medium with serum was linearly proportional to the culture time for HDFs.

**Figure 3:**
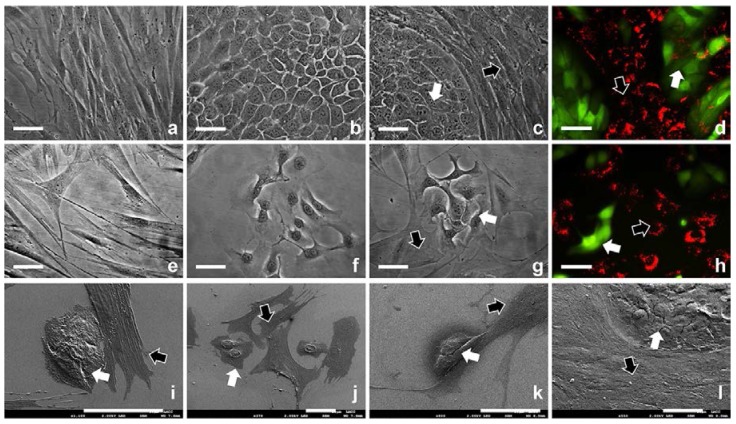
Micrographs of HDFs and HaCat cells mono- or co-cultured on tissue culture plastic (TCP) in medium with or without serum. Phase contrast micrographs of (**a**) HDFs, (**b**) HaCat cells, (**c**) HDFs and HaCat cells in medium with serum; (**e**) HDFs, (**f**) HaCat cells, (**g**) HDFs and HaCat cells in medium without serum. Fluorescent micrographs of HDFs and HaCat cells stained using RED and GREEN cell trackers respectively and co-cultured in medium (**d**) with or (**h**) without serum. Scanning electron microscopy (SEM) micrographs of HDFs and HaCat cells co-cultured in medium (**i**,**j**) without or (**k**,**l**) with serum. In the co-culture micrographs, HDFs and HaCat cells are pointed using WHITE and BLACK arrows respectively. Scale bar = 50 µm.

HDFs and HaCat cells were seeded onto TCP with different densities (5000, 10,000, 20,000, 40,000, 80,000, 160,000 cells/cm^2^ for mono-cultures, or the same cell densities with 1:1 ratio of both cell types for co-cultures) in medium with or without serum and cultured for 16 days. HaCat cells were observed to be less migratory and aggregated to form colonies, while HDFs were more migratory and behaved individually in both serum and serum-free cultures (Figure 3a,b,e,f). In serum-free medium HDFs cells were obviously less proliferative and more spread than in medium with serum (Figure 3a,e). Relatively more tightly packed colonies formed by less spread HaCat cells were observed in medium with serum in comparison with the more spread HaCat cells and loosely packed colonies in serum-free medium (Figure 3b,f). Population analysis (Table 1) indicated that all the HDFs mono-cultured in medium with serum became completely confluent within 1–7 days, while 66.9–100% confluent HaCat cells were obtained within 3–16 days, and the time to achieve the maximum confluence for both cell types was inversely proportional to the initial cell seeding densities. In serum-free medium, if the initial density was higher than or equivalent to 80,000 cells/cm^2^, 100% confluent HDFs and HaCat cells were achieved, and the time to reach the maximum confluence for both cell types was also inversely proportional to the initial cell seeding densities. However, if the initial density was lower than or equivalent to 40,000 cells/cm^2^, HaCat cells with significantly lower densities (0.4–7.1%) and HDFs with dramatically varying confluences (2.0–83.4%) were detected. When co-cultured in medium with or without serum, the HaCat colonies were surrounded by individual HDFs (Figure 3c,d,g–l). With the presence of serum, HDFs became approximately 59.8–69.6% confluent within 2–10 days, then gradually died out; while 100% confluent HaCat cells were obtained within 9–16 days if the cell seeding density of each cell type was higher than 5000 cells/cm^2^. For the lowest cell seeding density (2500 cells/cm^2^) investigated, approximately 67.4% confluent HaCat cells were achieved within 16 days, while 32.6% of the surfaces were still occupied by HDFs. Without the presence of serum, dramatically varying populations of both cell types were detected, and the confluences of HDFs (0.8–44.8%) and HaCat cells (0.1–100.0%) were heavily dependent on the cell seeding densities. When the initial densities of both cell types were higher or equivalent to 80,000 cells/cm^2^, completely confluent HaCat cells were achieved in medium without the supplemented serum.

### 2.2. 3D Cell Culture on Porous Substrates in Medium with or without Serum

**Figure 4:**
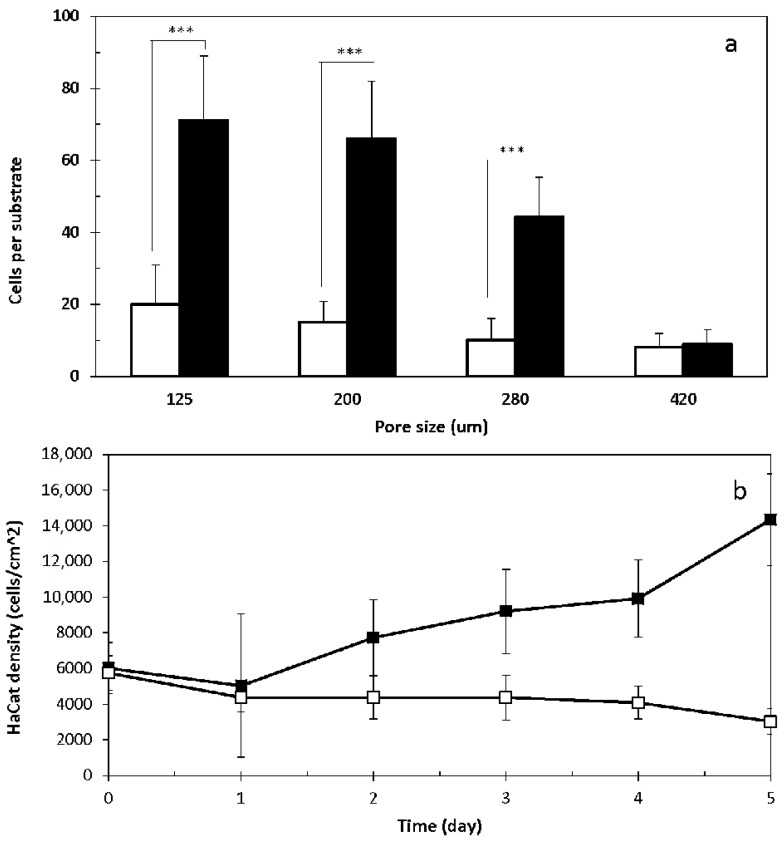
The influence of serum and HDF on the attachment of HaCat cells onto porous substrates. (**a**) HDFs stained with GREEN cell tracker were seeded onto modular substrates with open pores of different sizes (125, 200, 280, 420 μm) in medium without serum and incubated for 40 min. (**b**) HDFs seeded onto modular substrates with open pore size of 420 μm were cultivated in medium with (Black) or without (White) serum for 0 to 5 days, and then stained with GREEN cell tracker. HaCat cells stained with RED cell tracker were seeded onto modular substrates (**a**) with (Black) or without (White) pre-seeded HDFs in serum-free medium, (**b**) pre-cultured with HDFs in medium with or without serum, and incubated for 40 min. Fluorescent micrographs were captured, and analyzed via image J to register the attached HaCat cells. HDF and HaCat cell suspensions with the same volume (200 μL) and density (1 × 10^5^ cells/mL) were seeded onto each of these substrates. (Results shown are mean value ± SD, *n* = 3). (*** *p* < 0.001).

Aliquot of 200 µL HDF suspension (1 × 10^5^ cells/mL in serum-free medium and stained with GREEN cell tracker) was seeded onto each of the modular substrates with varying pore sizes (125, 200, 280 and 420 µm) fabricated in the 3D CCIS, and incubated for 40 min. The same volume (200 µL) of HaCat cell suspension (1 × 10^5^ cells/mL in serum-free medium and stained with RED cell tracker) was seeded onto each modular substrate with or without pre-seeded HDFs. After further incubation for 40 min, fluorescent micrographs of the attached cells were captured (Figure 1e–h). The total HaCat cell number on each substrate was registered and plotted against the pore sizes. As illustrated in Figure 4a, the open pore size demonstrated inverse impact on the number of HaCat cells attached onto the porous substrates with or without pre-seeded HDFs, and the presence of HDFs significantly enhanced the attachment of HaCat cells onto substrate with smaller open pores (125, 200 and 280 µm) but not with the largest pores (420 µm).

**Figure 5:**
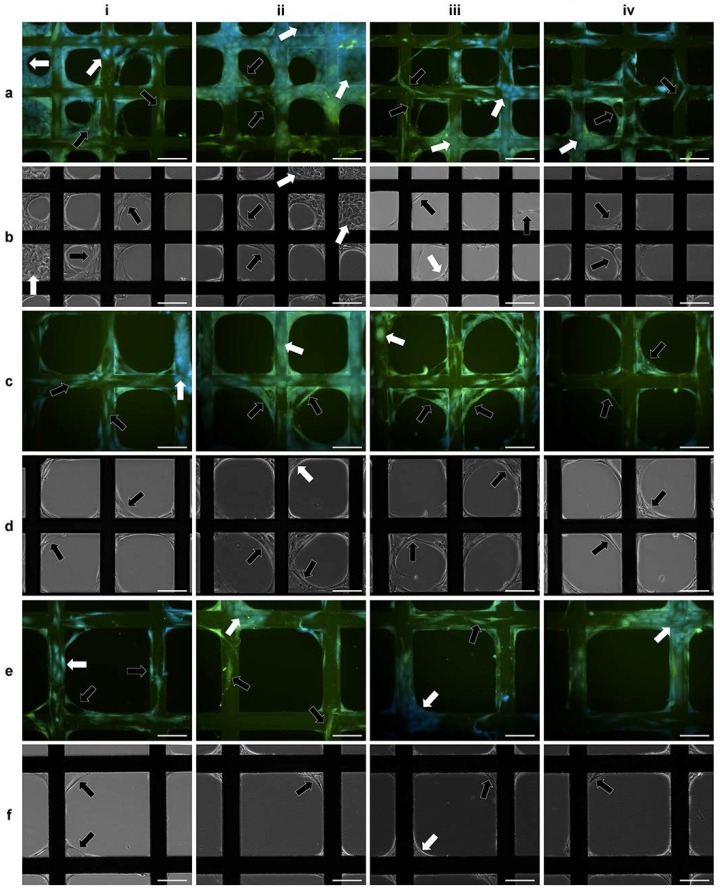
Micrographs of HaCat cells and HDFs co-cultured on porous modular substrates in serum-free medium. HDFs (200 μL, 1 × 10^5^ cells/mL) were seeded onto each of the modular substrates with pore sizes of (**a**,**b**) 125 μm, (**c**,**d**) 200 μm, (**e**,**f**) 280μm, and incubated for 40 min. HaCat cells (200 μL, 1 × 10^5^ cells/mL) were then seeded onto each of these substrates and cultured for 4 days. Fluorescent (**a**,**c**,**e**) and Phase contrast (**b**,**d**,**f**) micrographs were then captured. For fluorescent microscopy, all the cells were stained with GREEN cell tracker, and the cell nuclei were stained BLUE using DAPI. HDFs and HaCat cells are indicated using WHITE and BLACK arrows respectively. Scale bar = 100 μm.

In a separate experiment, HDFs (200 μL, 1 × 10^5^ cells/mL) were seeded onto modular substrates with the defined open pore size of 420 μm, incubated for 0–5 days in medium with or without serum, and then stained with GREEN cell tracker. HaCat cells (200 μL, 1 × 10^5^ cells/mL) stained with RED cell tracker were added onto each of these modular substrates and further incubated for 40 min. Fluorescent micrographs were captured (Figure 1i–l), and the numbers of the attached HaCat cells on per cm^2^ of substrate strut were registered. As shown in Figure 4b, the number of the attached HaCat cells was proportional to the culture period for HDFs in medium with serum. This was mainly due to the fact that HDFs colonized and bridged the open pores (Figure 1k–l), thus not only prevented the HaCat cells from filtrating through the porous substrates, but also acted as the extra cell substrates for the HaCat cells to attach onto. The influence of HDF culture time on HaCat cell attachment was not obvious in the serum-free environment, as significantly less HDFs were able to colonize and bridge the porous substrates with large open pores (420 μm). To further investigate the co-culture of these two cell types on porous substrates in medium without serum, HDFs (200 μL, 1 × 10^5^ cells/mL) were seeded onto each of the substrates with different pore sizes (125, 200 and 280 µm) and incubated for 40 min. This was followed by the additional seeding of HaCat cells (200 μL, 1 × 10^5^ cells/mL) onto each of these substrates. After further co-cultured in serum-free medium for 3–4 days, both cell types were observed to completely colonize within some of the smallest pores (125 μm) as shown in Figure 5ai–ii,bi–ii, but not within any of the larger pores (i.e., 200 and 280 μm) (Figure 5).

### 2.3. 3D Cell Culture on Cellulosic Scaffolds in Medium with or without Serum

**Figure 6:**
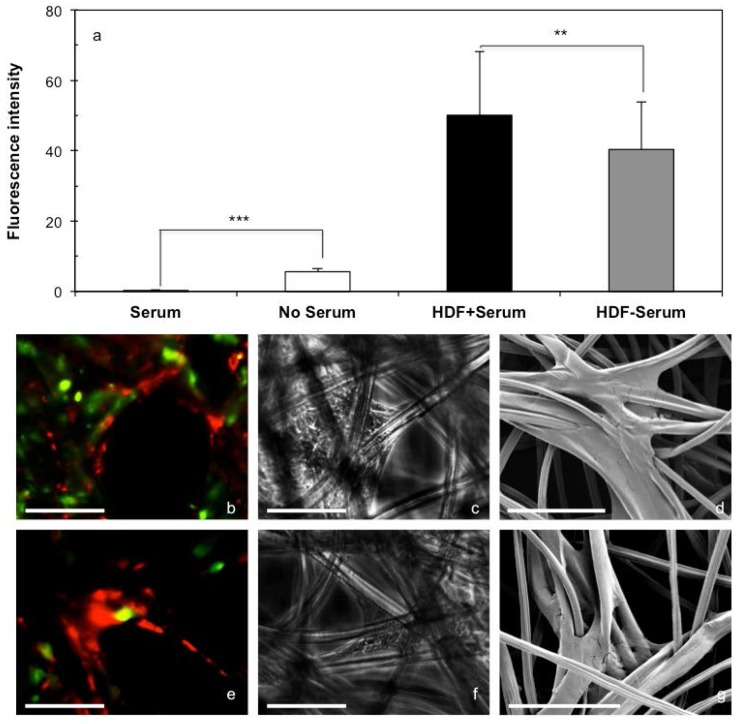
The influence of serum and HDFs on the attachment of HaCat cells onto cellulosic scaffolds. HDFs stained with RED cell tracker were seeded onto the cellulosic scaffolds (2 × 10^5^ cells/cm^2^ of scaffold) and cultured for 0 or 6 days in medium with or without serum. HaCat cells stained with GREEN cell tracker were then seeded onto the scaffolds (1 × 10^6^ cells/cm^2^) with or without HDFs in medium with or without serum, and incubated for 24 h. (**a**) Fluorescent intensities of the attached HaCat cells on the scaffolds with or without pre-seeded HDFs in medium with or without serum were quantified. Fluorescent (**b**,**e**), phase contrast (**c**,**f**) and SEM (**d**,**g**) micrographs of the HaCat cells attached onto the scaffolds pre-cultured with HDFs in medium (**b**–**d**) with or (**e**–**g**) without serum were captured. Results shown are mean ± SD (*n* = 3). (** *p* < 0.01, *** *p* < 0.001). Scale bar = 100 µm.

HDFs stained with RED cell tracker were seeded onto the cellulosic scaffolds (2.0 × 10^5^ cells/cm^2^) in medium with or without serum and divided into 2 groups. In the 1st group, HaCat cells stained with GREEN cell tracker were seeded onto the scaffolds (1.0 × 10^6^ cells/cm^2^) with or without pre-seeded HDFs in medium with or without serum. After further incubation for 24 h, fluorescent micrographs of both cell types on the scaffolds were captured for fluorescent intensity quantification. As shown in Figure 6a, more HaCat cells attached onto the cellulosic scaffolds in medium without serum than with serum, while the presence of HDFs also detectably facilitated the attachment of HaCat cells in medium with or without serum. In the 2nd group, the HDFs seeded on the cellulosic scaffolds were cultured for 6 days, and the cell-scaffold composites were seeded with HaCat cells (1.0 × 10^6^ cells/cm^2^) that were stained with GREEN cell tracker, further cultured for 24 h and then analyzed using different microscopic technologies. Fluorescent micrographs (Figure 6b,e) clearly demonstrated the complex structures formed by HDFs within the porous scaffolds in medium with or without serum, which not only prevented HaCat cells from passing through the open pores, but also acted as the extra cell substrates for HaCat cells to attach onto. The complex cell structures were also confirmed by both PCM (Figure 6c,f) and SEM micrographs (Figure 6d,g).

**Figure 7:**
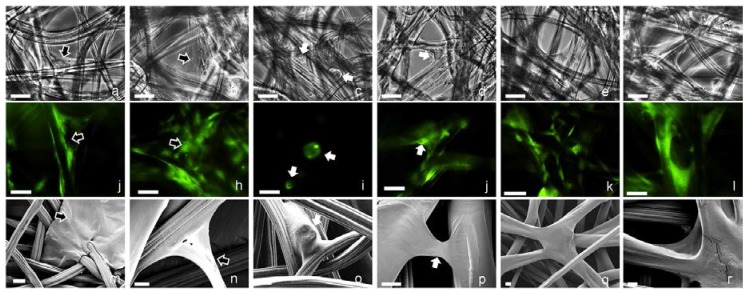
Micrographs of HDFs and HaCat cells cultured on the cellulosic scaffolds in medium with or without serum. (**a**–**f**) Phase contrast, (**j**–**l**) fluorescent, (**m**–**r**) SEM micrographs of the scaffolds cultured with HDFs in medium (**a**,**j**,**m**) with or (**b**,**h**,**n**) without serum; HaCat cells in medium (**c**,**i**,**o**) with or (**d**,**j**,**p**) without serum; both HDFs and HaCat cells in medium (**e**,**k**,**q**) with or (**f**,**l**,**r**) without serum. For fluorescent microscopy, all the cells were stained using GREEN cell tracker. HDFs and HaCat cells in the mono-cultures are indicated using BLACK and WHITE arrows respectively. Scale bars: (**a**–**l**) 50 µm, (**m**–**r**) 10 µm.

**Figure 8:**
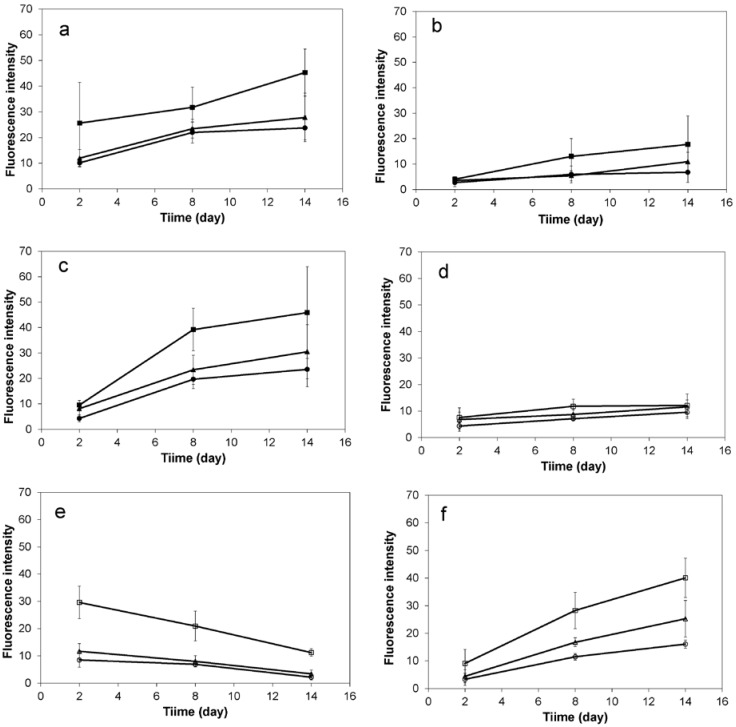
The influence of serum, cell seeding density and culture period on the populations of HDFs and HaCat cells mono- or co-cultured on cellulosic scaffolds. (**a**,**d**) HDFs, (**b**,**e**) HaCat cells, or (**c**,**f**) both HDFs and HaCat cells with 1:1 ratio, were seeded onto the scaffolds with different densities (○/●: 1.0 × 10^5^, Δ/▲: 1.5 × 10^5^, □/■: 2 × 10^5^ cells per cm^2^ of the scaffolds) and cultured in medium (**a**–**c**) with (Black) or (**d**–**f**) without (White) serum. All the cells were stained with GREEN cell tracker and imaged at days 2, 8 and 14; the cell populations in the scaffolds were estimated by analyzing the percentages of the fluorescent areas in each micrograph via Image J. Results shown are mean ± SD (*n* = 3).

HDFs and HaCat cells were seeded onto the cellulosic scaffolds (1.0 × 10^5^, 1.5 × 10^5^, 2.0 × 10^5^ cells per cm^2^ of the scaffold for mono-cultures, and the same cell densities with 1:1 ratio of both cell types for co-cultures), and cultured in medium with or without serum for 2 weeks. HDFs demonstrated very similar behaviors in medium with or without serum, as they were observed to attach onto the cellulosic fibers with elongated morphologies and bridge the open pores by forming complex structures as shown in Figure 7a,b, which was confirmed by fluorescent (Figure 7j,h) and SEM micrographs (Figure 7m,n). In comparison, HaCat cells demonstrated very different behaviors in medium with or without serum. With the presence of serum, HaCat cells aggregated to form cell spheres of varying sizes when initially seeded onto the cellulosic scaffolds as shown in Figure 7c,i,o. Without the presence of serum, the majority of HaCat cells attached onto the fibers and no cell aggregate was observed (Figure 7d,j,p). When co-cultured on the scaffolds, there was no HaCat cell aggregation detected. As culture progressed, both cell types interacted with each other to form complex structures within the open pores in medium with or without serum, but it was difficult to distinguish and identify each cell type due to the complex structures of the natural scaffolds (Figure 7e,f,k,l,q,r). As shown in Figure 8a,d, the fluorescence intensity of HDFs mono-cultured onto the scaffolds was dependent on the culture period in medium with or without serum, and less cells were detected in serum-free medium than in medium with serum. When HaCat cells were cultivated in the natural scaffolds, more cells were detected initially in medium without serum than with serum. However, as culture continued the cell population gradually increased in medium that contained serum, but decreased in medium without supplemented serum (Figure 8b,c). When co-cultured on the cellulosic scaffolds, the total population of both cell types was proportionally dependent on the initial cell seeding density and the culture period; and comparable cell populations were achieved in medium with or without supplemented serum (Figure 8c,f).

## 3. Discussion

Since the underpinning biological processes during tissue formation are complex, and still comprise multiple known as well as unknown factors [23,24], it is a challenging task to further advance the current PoC level studies in tissue engineering to manufacture functional cells or tissues for clinical applications. This study aimed to closely integrate 3 different cell and tissue culture technologies as a generic TE research platform not only for the in-depth understanding of skin cells in different culture environments, but also for the design of more suitable cell seeding and culturing strategies in serum-free medium for clinical purposes.

From the 1980s to the 1990s, 2D cell culture was used to investigate fibroblast attachment onto tissue culture surfaces, the presence of serum components was reported to adversely influence cell adhesion [25], which has been confirmed by the HDF culture experience in serum-free medium in our research group [16,17]. In this study, we demonstrated that serum depletion facilitated the attachment of HaCat cells onto TCP, which was further enhanced by the presence of HDFs. Due to the complex interactions between fibroblasts and keratinocytes, HDFs might facilitate HaCat cell attachment through multiple mechanisms including soluble factors, extracellular matrix and/or direct cell-cell contacts as previously reported [26,27]. When cultured on TCP in medium with or without serum, HDFs behaved individually and retained typical spindle-like morphologies, while HaCat cells tended to form colonies and their proliferation was dependent on both serum and cell seeding densities. As the keratinocyte colonies formed within epidermis, epidermal appendages, or recreated in 2D cell cultures, are subject to auto-regulation [28,29], the requirement of HaCat cells on high seeding density for colony formation and active proliferation can be explained as the genetically definable quantitative trait [30]. Within slow growing colonies, keratinocytes were observed to pack loosely, this could lead to the adjacent keratinocytes not forming the necessary coherence through the desmosomal junctions [31]; while the successful culture using high initial densities could be due to the short distances and extensive intercellular junctions between the cultivated keratinocytes [32]. The mono-cultured HDFs and HaCat cells were less proliferative in serum-free medium due to the depletion of the growth factors in serum [10,11]. When co-cultured, however, both HDFs and HaCat cells could populate in medium without serum due to the dynamic cell-cell interactions [33,34]. The populations of both cell types in serum-free medium were also dependent on high initial cell densities and relatively longer culture periods, which can be explained as the necessity of sufficient cell-cell interactions and the accumulation of extracellular matrix proteins and critical growth factors [35].

Cell seeding experiments using the scale-down model indicated that both serum depletion and the pre-seeded HDFs enhanced the attachment of HaCat cells onto the modular substrates, which confirmed the observations in 2D cultures. Moreover, the HDFs pre-cultured in the scale-down model were observed to segment, bridge and even totally seal the finely controlled open pores by forming complex structures; the number of the subsequently seeded HaCat cells that filtrated through the porous substrates was thus reduced dramatically. This seemed to be an additional mechanism for HDFs to facilitate HaCat attachment in the scale-down model, thus pre-culture of HDFs on the porous substrates can be used as an alternative method to increase the seeding efficiency of HaCat cells. Even though both HDFs and HaCat cells co-cultured in the scale-down model with serum-free medium might still dynamically interact with each other [33,34,35], both cell types were only able to fully colonize and occupy the small pores (125 µm), but not the larger pores (200, 280, 400 µm). This was mainly due to the relatively low initial cell density on the modular substrates with large pores, as the number of cells attached onto the substrates was observed to be inversely dependent on the pore sizes.

When seeded onto the cellulosic scaffolds in medium with serum, HaCat cells were observed to aggregate instead of attaching onto the scaffold; while serum depletion demonstrated significant influence on the attachment of HaCat cells onto these scaffolds as shown in both 2D cultures and the scale-down model. The TCPs, the modular substrates and the cellulosic scaffolds were not pre-coated with any proteins in this research, hence the adhesion of HaCat cells onto these materials in serum-free medium was not facilitated by any coated proteins through the well-established integrin-ligand interaction mechanism [36,37]. Since multivariate biological activities are significantly influenced by the wettability or the properties of the structured water on material surfaces [38,39], the adhesion of HaCat cells on very different materials observed in this research might be directly induced by the structured water but not the materials [40,41]. As shown in 2D culture and the scale-down model, the pre-seeded HDFs were demonstrated to facilitate HaCat cell adhesion onto the cellulosic scaffolds, suggesting the consistency of these 3 culture systems. The complex HDF structures formed in the cellulosic scaffolds also prevented the subsequently seeded HaCat cells from passing through the open pores, thus the pre-cultured HDFs can be used as an additional mechanism to increase the HaCat cell seeding efficiency in this tissue culture system. The observations clearly suggested very different strategies (i.e., serum depletion, pre-seeded HDFs or pre-cultured HDFs) that can be used to achieve high initial densities of HaCat cells with the porous substrates and scaffolds. When both HDFs and HaCat cells were seeded with high densities and co-cultured on the cellulosic scaffolds, comparable cell populations were achieved in medium with or without serum. Apart from the dynamic interactions between these 2 skin cell types [33,34,35], it might be also due to the existence of both small and large open pores within a very broad range (50–300 µm), so the necessary high cell densities could be achieved within the cellulosic scaffolds. Considering the cost and more importantly the clinical issues associated the use of serum [12,13], successful culture of skin cells in serum-free media is critically valuable for clinical applications.

Overall, our cell and tissue culture experiments via the generic platform illustrated both the consistency and the discrepancy between 2D and 3D culture technologies. Firstly, it was demonstrated that HDFs and HaCat cells maintained certain inherent characteristics in all cultures. For example, the adhesion of HaCat cells onto 3 different materials were all facilitated by serum depletion and pre-seeded HDFs; cell proliferations in these culture systems with serum-free medium were all dependent on high initial cell densities. Secondly, very distinct cell behaviors were detected in three different culture systems. As complex HDF structures were observed only in 3D culture systems but not in 2D cultures, the pre-cultured HDFs could be used as an additional strategy to increase the seeding efficiency of HaCat cells for 3D cultures. This study suggested that 2D cell culture is still a convenient, valuable, and high throughput technology for the investigation of inherent cellular properties and essential cell-cell interactions that exist in all three culture systems. Even though the *in vitro* tissue culture model is not ideal for the mechanistic understanding of tissue formation [21,42], but it is valuable for the identification and evaluation of the major process variables in tissue manufacturing processes. For the ease of microscopic analysis of cell-cell and cell-scaffold interactions during tissue culture, single layered thin substrates with finely controlled open pores were used in the scale-down model. Due to the change of multiple experimental variables between the scale-down and 3D models, direct comparison of each specific variable (e.g., cell seeding density, surface chemistry, mechanical strength and stiffness etc.) is still difficult. Hence more research efforts to standardize the scale-down and 3D models using the same material or surface chemistry are necessary. However, the simplification of the complex structure of the porous scaffold in the scale-down model has enabled us to recognize and explain some of the similarities and differences between 2D cell and 3D tissue cultures. Therefore, the development of the generic research platform has made it feasible to compare and analyze some of the experimental results from very distinct culture systems. Due to the mechanistic insights of 2 skin cell types obtained from the generic platform, more suitable cell seeding and culturing strategies in serum-free medium can be designed to address the clinical challenges associated with the use of serum in skin tissue engineering.

## 4. Materials and Methods

### 4.1. Cell Culture

Neonatal foreskin human dermal fibroblasts (HDFs, Intercytex, Manchester, UK) and immortalized human keratinocytes (HaCat cells, Addexbio, San Diego, CA, USA) were cultured in Dulbecco’s modified Eagle’s medium (DMEM, Lonza, Slough, UK) containing 4.5 g/L glucose; supplemented with 2 mM l-glutamine (Sigma, Dorset, UK), 100 IU/mL penicillin and 100 μg/mL streptomycin (Sigma, Dorset, UK), and 10% (*v*/*v*) fetal bovine serum (FBS, Fisher Scientific, Loughborough, UK). Cells were cultivated in T-flasks at 37 °C in 5% CO_2_ humidified atmosphere. Media in the flasks were changed every three days and the cells were continually passaged prior to experimentation at 80–90% confluence using trypsin/EDTA (0.02% (*w*/*v*) solution). The medium without supplemented FBS was also prepared for serum-free cell culture experiments.

### 4.2. Cellulosic Scaffolds and Modular Substrates for 3D Cell Culture

As cellulose is a commonly used natural biomaterial for diverse research and medical applications [43,44,45], a commercially available porous cellulosic scaffold (Azowipes^®^: non-woven viscose rayon bonded with a styrene buta-diene copolymer, Vernon-Carus Ltd., Wiltshire, UK) was used to culture both HDFs and HaCat cells. The fibers of this natural scaffold were 20–50 µm in diameter, and the open pores were 50–300 µm in diameter. The thickness of each scaffold layer was 0.1–0.2 mm and single layers were used for 3D tissue culture in this research. All the cellulosic scaffolds were thoroughly washed in Phosphate-buffered saline (PBS) and DMEM medium to remove all proprietary alcohol-based solvents in which they were soaked prior to cultivation of cells as previously described [22].

Commercially available TEM nickel specimen supporters (Diameter: 3.05 mm, thickness: 10–30 µm, strut bar width: 40–80 µm, Agar Scientific, Stansted, UK) with finely controlled square meshes of different sizes (125, 200, 280 and 420 µm) were utilized as the modular porous substrates in the 3D CCIS. After washed thoroughly using distilled water, dried and autoclaved, the thin modular substrates were then suspended in the 3D CCISs. The cell seeding method for the 3D CCIS was previously described [22].

### 4.3. Phase Contrast Microscopy

The cells cultured on the surfaces of TCP, cellulosic scaffolds and suspended modular porous substrates were monitored and analyzed noninvasively using an inverted Phase-Contrast Microscope (Nikon Eclipse Ti) during culture. Cell orientations and outgrowths were determined from the micrographs using image analysis software (NIS-Elements). Time-lapse videos of the cells were captured during cell culture using the Bio Station (Nikon Bio Station CT). Image analysis was also conducted using Image J software (Image J).

### 4.4. Live Cell Fluorescent Microscopy

The cells cultured on TCP, cellulosic scaffolds and suspended modular substrates were labeled with Cell Tracker™ RED (CellTracker™ CM-DiI Dye) or GREEN (CellTracker^TM^ Green CMFDA Dye, Thermo Fisher Scientific, Paisley, UK) for live cell fluorescent microscopy during cell culture. An aliquot of 5 or 0.2 µL of RED or GREEN Cell Tracker™ stock solution prepared in DMSO was directly added into 1 mL of the cell culture medium to the working concentrations (5 and 0.5–25 µM respectively). After incubated for 1–24 h at 37 °C and 5% CO_2_, the medium with Cell Tracker^TM^ reagent was replenished with fresh medium. Cell culture was then continued and the labeled cells were imaged and analyzed using a fluorescent microscope (Nikon Eclipse Ti) at λ_ex_ = 553 nm, λ_em_ = 570 nm (for FITC/GREEN Cell Tracker™ visualization), and at λ_ex_ = 580 nm, λ_em_ = 650 nm (for TRITC/RED Cell Tracker™ visualization) respectively. Fluorescence microscopy was performed after culture to determine the location of the attached cells on the porous substrates by staining the nuclei with DAPI (300 nM, Vector Laboratories Inc., Burlingame, CA, USA). In brief, after removal of the culture medium, the cells cultured on the modular substrates were washed gently with PBS (×3). The cells were fixed in IC Fixation Buffer (Fisher Scientific, Loughborough, UK) for 10 min, and labeled with DAPI for 10 min. After a final wash (×3 PBS), fluorescence images of the cells were captured at λ_ex_ = 358 nm, λ_em_ = 461 nm (for DAPI/nuclei visualization).

### 4.5. Scanning Electron Microscopy

The cells cultivated on TCP, cellulosic scaffolds, and free-standing modular substrates were gently washed with PBS (×3), fixed in IC Fixation Buffer (Fisher Scientific, Loughborough, UK) for 20 min, then washed with distilled water, left to dry at room temperature, and coated with gold and palladium (80:20 ratio) using a splutter coater (Quorum Q150R S, Laughton, UK). In situ analysis of the cells was then conducted via SEM (JSM 7800F, JEOL, Peabody, MA, USA).

### 4.6. Statistics

Student’s unpaired t-test was used to compare the seeded or cultivated cells in TCP, cellulosic scaffolds or modular substrates in medium with the presence or absence of serum (* *p* < 0.05, ** *p* < 0.01, *** *p* < 0.001).

## Figures and Tables

**Table 1:** The influence of serum, cell-seeding density and culture time on the populations of HDFs and HaCat cells mono- or co-cultured on tissue culture plastic (TCP). Results shown are mean ± SD (*n* = 3).

Cell	Density (Cells/mL)	With Serum		Without Serum	
		Maximum Confluence (%)	Time to Achieve Confluence (Day)	Maximum Confluence (%)	Time to Achieve Confluence (Day)
MONO-HDF	5000	100.00	7	2.02	2
	10,000	100.00	6	3.27	2
	20,000	100.00	5	25.07	8
	40,000	100.00	3	83.44	8
	80,000	100.00	2	100.00	6
	160,000	100.00	1	100.00	2
MONO-HaCat	5000	66.99	16	0.42	1
	10,000	91.50	16	0.61	1
	20,000	100.00	10	1.36	2
	40,000	100.00	8	7.11	6
	80,000	100.00	7	100.00	9
	160,000	100.00	3	100.00	3
CO-HDF	2500	60.91	12	0.78	1
	5000	60.10	10	1.35	2
	10,000	66.01	8	7.34	5
	20,000	66.36	6	26.80	7
	40,000	69.61	4	39.42	5
	80,000	59.87	2	44.87	3
CO-HaCat	2500	67.42	16	0.11	1
	5000	100.00	16	0.13	1
	10,000	100.00	14	0.47	1
	20,000	100.00	12	1.41	2
	40,000	100.00	10	28.59	8
	80,000	100.00	9	100.00	9
